# Long non-coding RNA SNHG3 promotes progression of gastric cancer by regulating neighboring MED18 gene methylation

**DOI:** 10.1038/s41419-019-1940-3

**Published:** 2019-09-18

**Authors:** Yi Xuan, Yanong Wang

**Affiliations:** 1 Department of Gastric Surgery, Fudan University Shanghai Cancer Center, Shanghai, 200032 China; 2 Department of Oncology, Shanghai Medical College, Fudan University, No 270 Dongan Road, Xuhui, Shanghai 200032 China

**Keywords:** Cancer, Cancer

## Abstract

To understand the mechanistic involvement of long non-coding RNA (lncRNA) SNHG3 in gastric cancer (GC), the relative abundance of SNHG3 was determined by real-time PCR. Overall and metastasis-free survival was analyzed by Kaplan–Meier’s plot. The potential impact of SNHG3 on tumor progression was evaluated both in vitro and in vivo. The in vivo metastasis was monitored in the tail vein-injected mice. Our data suggested that high SNHG3 associated with unfavorable prognosis in respect to overall and metastasis-free survival. SNHG3-deficiency significantly suppressed cell proliferation and cell viability in vitro and xenograft progression in vivo. In addition, ectopic overexpression of SNHG3 promoted cell migration and invasion in vitro and lung metastasis in vivo. Mechanistically, we uncovered SNHG3 associated with EZH2 and negatively regulated MED18 expression through methylation modulation. Transient knockdown of MED18 in SNHG3-deficient cells completely rescued the tumor suppressive phenotypes in GC cells. Our data unraveled the oncogenic properties of high SNHG3 in GC, which predominantly depended on epigenetically regulated MED18.

## Introduction

Gastric cancer (GC) is one of the most common human malignancies worldwide. It’s estimated that 950,000 new cases were diagnosed and around 723,000 deaths were claimed by this disease in 2012^1^. The most common cause of GC is infection by the Helicobacter pylori, which accounts for more than half of the morbidity. The other well-recognized risk factors include cigarette smoking, dietary intake of pickled vegetables and obesity^2^. Approximately 1–3% of GC incidence are linked to the inherited genetic aberrances^3^. Diagnosis of GC is usually by biopsy performed during endoscopy examination^4^. Clinical managements of GC majorly include surgery, chemotherapy, radiation therapy and targeted therapy^5^. Early diagnosis is extremely critical for the curative purpose in those patients at early stage of disease progression. Globally, the overall outcomes of GC are relatively poor with a <10% five-year survival rate^6^. The precise medicine is still in urgent need based on the comprehensive understanding of the biology underlying this disease.

Long non-coding RNA (lncRNA) is a class of transcripts longer than 200 nucleotides without evident protein coding potential^7^. Increasing knowledges have uncovered kaleidoscopic biological functions of lncRNAs in microorganisms, plants, animals and human. Notably, accumulative evidences disclosed the indispensable involvement of lncRNAs in human malignancies in either pro-tumoral or anti-tumor manners^8^.

Small Nucleolar RNA Host Gene 3 (SNHG3) is a novel lncRNA potentially associated with Alzheimer disease and colorectal cancer. For instance, Zhang et al. demonstrated that in hepatocellular carcinoma SNHG3 expression correlated with the malignant status and relatively poorer prognosis^9^. Huang et al. identified SNHG3 as a competing endogenous RNA molecule to promote malignant progression of colorectal cancer^10^. Hong et al. characterized aberrantly up-regulated SNHG3 in ovarian cancer, which intimately associated with unfavorable prognosis and enhanced malignant progression^11^. Consistently, the study performed by Li et al. disclosed that SNHG3 regulated energy metabolism of ovarian cancer via a systematic analysis of mitochondrial proteomes^12^. In glioma, Fei et al. found that SNHG3 enhanced the malignant behaviors through specifically silencing KLF2 and p21^13^. LncRNA SNHG3 also was characterized to be involved in the microRNA pathway in hepatocellular carcinoma, wherein the modulation of miR-128/CD151 signaling by SNHG3 induced epithelial-mesenchymal transition (EMT) and sorafenib resistance^14^. More recently, Liu et al. proposed that up-regulation of SNHG3 promoted lung adenocarcinoma proliferation as well^15^. Despite of the well-recognized oncogene roles of SNHG3 in array of human cancers, the mechanistic involvement of SNHG3 in GC is still elusive. To clarify this issue, here we set out to analyze relative expression of SNHG3 transcripts in GC both in vitro and in vivo. Potential contribution to the tumor biology of SNHG3 in this disease was also systematically interrogated. Furthermore, we attempted to understand the fundamental molecular mechanisms behind the oncogenic properties of this lncRNA.

## Materials and methods

### Cell culture

Normal gastric cell lines GES-1 (epithelial cell derived from human gastric mucosa) and tumor cell lines including MGC-803, AGS, BGC-823, SGC-7901, MKN-45, and HGC-27 were ordered from the Chinese Academy of Medical Science. All cells were maintained in modified RPMI medium (Hyclone, MA, USA) in humidified incubator containing 5% CO_2_. The culture medium was supplemented with 10% fetal bovine serum (FBS, Gibco, MA, USA), 100 U/mL penicillin and 100 μg/mL streptomycin (Gibco). Cell transfection was achieved with Lipofectamine 3000 (Invitrogen, MA, USA) following the manufacturer’s guide. The following shRNA sequenced were constructed for gene silencing purpose:

SNHG3 shRNA1: 5′-GGGCACTTCGTAAGGTTTAAA-3′

SNHG3 shRNA2: 5′-GGTTGAGTGCAAGATGAGTTA-3′

MED18 siRNA: 5′-AGCTTAGCCGTTACGCGA-3′

Stable SNGH3-deficient MKN-45 was established by lentivirus infection followed by puromycin selection.

### Quantitative real-time PCR

Trizol kit (Invitrogen, MA, USA) was employed to extract total RNA from either cells or tissues. Reverse transcription was conducted with the commercially available kit (Takara, Dalian, China). The SYBR Green MasterMix (Takara, Dalian, China) was used for qPCR reaction on ABI 7900 system. The expression of SNHG3 was calculated using 2^-∆∆Ct^ method and endogenous GAPDH was used for internal reference. The primer sequences were provided as below:

SNHG3 forward primer: 5′-TTCAAGCGATTCTCGTGCC-3′

SNHG3 reverse primer: 5′-AAGATTGTCAAACCCTCCCTGT-3′

GAPDH forward primer: 5′-ACAACTTTGGTATCGTGGAAGG-3′

GAPDH reverse primer: 5′-GCCATCACGCCACAGTTTC-3′

MED18 forward primer: 5′-AGAATCGCTTGAACCCAGGA-3′

MED18 reverse primer: 5′-AGTTTCGCTCTTCTCACCCA-3′.

MED18 promoter forward primer: 5′-ACTTCTCAGAGCCTATTTCC-3′

MED18 promoter reverse primer: 5′- ACTTACGAAGGGTGGACAT-3′

### CCK-8 assay

The proliferation of MKN-45 and SGC-7901 cells was measured using the Cell Counting Kit-8 (CCK-8, Dojindo, Japan). Briefly, 2 × 10^3^ cells were seeded into 96-well plate in triplicate and cultured for 24 h at 37 °C. The OD 450 nm was recorded by microplate reader following the manual of CCK-8 kit.

### Colony formation assay

The indicated cells were digested and seeded into 96-well plates for 7-days continuous culture. The cells were then fixed with 4% PFA and stained with 0.5% crystal violet for 10 min. The colonies were counted in three independent fields under light microscope and representative images were captured.

### Tumor xenograft experiment

Athymic BALB/c mice (5 weeks) were purchased from the Vital River Labrotary (Beijing, China) and maintained in SPF environment. The animal study was conducted strictly following the protocol approved by the Institutional Ethics Committee of Fudan University Shanghai Cancer Center and under the Guide for the Care and Use of Laboratory Animals by NIH. Briefly, SNHG3-proficient SGC-7901 cells were subcutaneously inoculated into the lower right flank of nude mouse. Tumor growth was regularly monitored and volume was calculated as 0.5 × length × width^2^. The tumor-bearing mice were sacrificed after 30 days and xenograft tumors were excised.

### Wound healing

Indicated cells were trypsinized and seeded into 6-well plate in triplicate. After 24 h, the scratch was created using sterile pipette tips when the cell confluency above 90%. Cells were maintained in serum-free medium and healing processing was recorded with inverted microscope, and the migration was measured as the percentage of wound width.

### Transwell assay

Indicated cells were re-suspended in serum-free RPMI medium to a concentration of 1 × 10^5^/mL, and 100 μL were added into the upper chamber of matrigel-precoated (BD BioSciences, CA, USA) transwell inserts (Corning, NY, USA). The lower compartment was supplemented with complete RPMI medium. After 12 h of incubation, cells free in the upper chamber were wiped off with cotton swab. The invaded cells were fixed and stained with 0.25% crystal violet, the typical images were captured and invaded cells were counted in five random fields under the microscope.

### In vivo metastasis assays

To investigate metastasis in vivo, MKN-45 cells carrying either sh-CTR, shSNHG3-1 or shSNHG3-2 were administrated into nude mice (1 × 10^6^ cells/100 μL) via tail vein injection. The subject mice were terminated 10 days post-injection. The lung was excised and subjected to HE staining. The metastatic foci were examined histologically.

### Western blot assay

Equal amounts of protein (20 μg) were resolved by electrophoresis with 10% polyacrylamide gels, and followed by PVDF membrane transferring (Millipore, MO, USA) in ice bath. The PVDF membranes were examined with specific primary antibodies (anti-MED18, sc-514415, 1:1000; anti-vimentin C-20, sc-7557, 1:1000; Santa Cruz Biotechnology, CA, USA; anti-SNAI1, ab117866, 1:1000, Abcam, Cambridge, UK; anti-E-cadherin 24E10, #3195, 1:1000; anti-β-actin, #3700, 1:1000, Cell Signaling Technology, MA, USA) for 1 h at ambient temperature. The incubation with HRP-conjugated secondary antibody (anti-mouse, 1:5000, #7074, Cell Signaling Technology, MA, USA) was performed at R.T. for 1 h. The enhanced chemiluminescent substrate (Millipore, MO, USA) was used to visualize the target bands. Quantitation was conducted by densitometry scanning and calculated with ImageJ software.

### Subcellular localization of SNHG3

Fractioning was performed with PARIS Kit (Life Technologies, CA, USA) to separate nuclear and cytoplasm. The U6 and GAPDH transcripts were used as markers to evaluate the fractioning efficiency. SNHG3 contents were measured in the indicated fractions by real-time PCR.

### RNA immunoprecipitation (RIP)

RIP assay was conducted using EZ-Magna RIP kit (Millipore, MO, USA) in accordance with the provider’s guide. The anti-EZH2 antibody (Abcam, Cambridge, UK) and control IgG was used. The RNA species in EZH2 immunoprecipitate complex was measured by qPCR.

### Chromatin immunoprecipitation (ChIP) assay

The EZ-Magna ChIP TMA Kit (Millipore, MA, USA) was used to investigate the potential association between EZH2 and MED18 promoter in accordance with the manufacturer’s manual. Chromatin was crosslinked by formaldehyde treatment and ultrasonicated to generate fragments with average length of 200–300 bp. DNA fragments co-immunoprecipitated with EZH2 antibody was further analyzed by qPCR. The IgG antibody served as control. The probe sequences used in our assay were provided below:

Forward: 5′-ACTTCTCAGAGCCTATTTCC-3′

Reverse: 5′-ACTTACGAAGGGTGGACAT-3′.

### Bisulfite sequencing PCR (BSP)

DNA was extracted from GC cell lines with the DNeasy. Blood and Tissue Kit (Qiagen, CA, USA) and followed by bisulfate modification. The bisulfite sequencing analysis was performed with the EpiTect Bisulite Kit (Qiagen, CA, USA) following the provider’s manual. The following primers were used for PCR: methylated MED18 forward: 5′-GTTAAGGATTTAGGTAGCGTTCG-3′, reverse: 5′-GTTCCCAAACGTATAAATACCGA-3′; unmethylated MED18 forward: 5′- AAGGATTTAGGTAGTGTTTGGAG-3′, reverse: 5′- CATTCCCAAACATATAAATACCAAAA-3′.

### Statistical analysis

All data were expressed as Mean ± SD. Statistical comparison was analyzed using the Student *t* test or two-way ANOVA method followed by a Tukey’s post hoc test. *P* < 0.05 was considered as significantly different.

## Results

### High SNHG3 in GC associated with poor prognosis

**Fig. 1:**
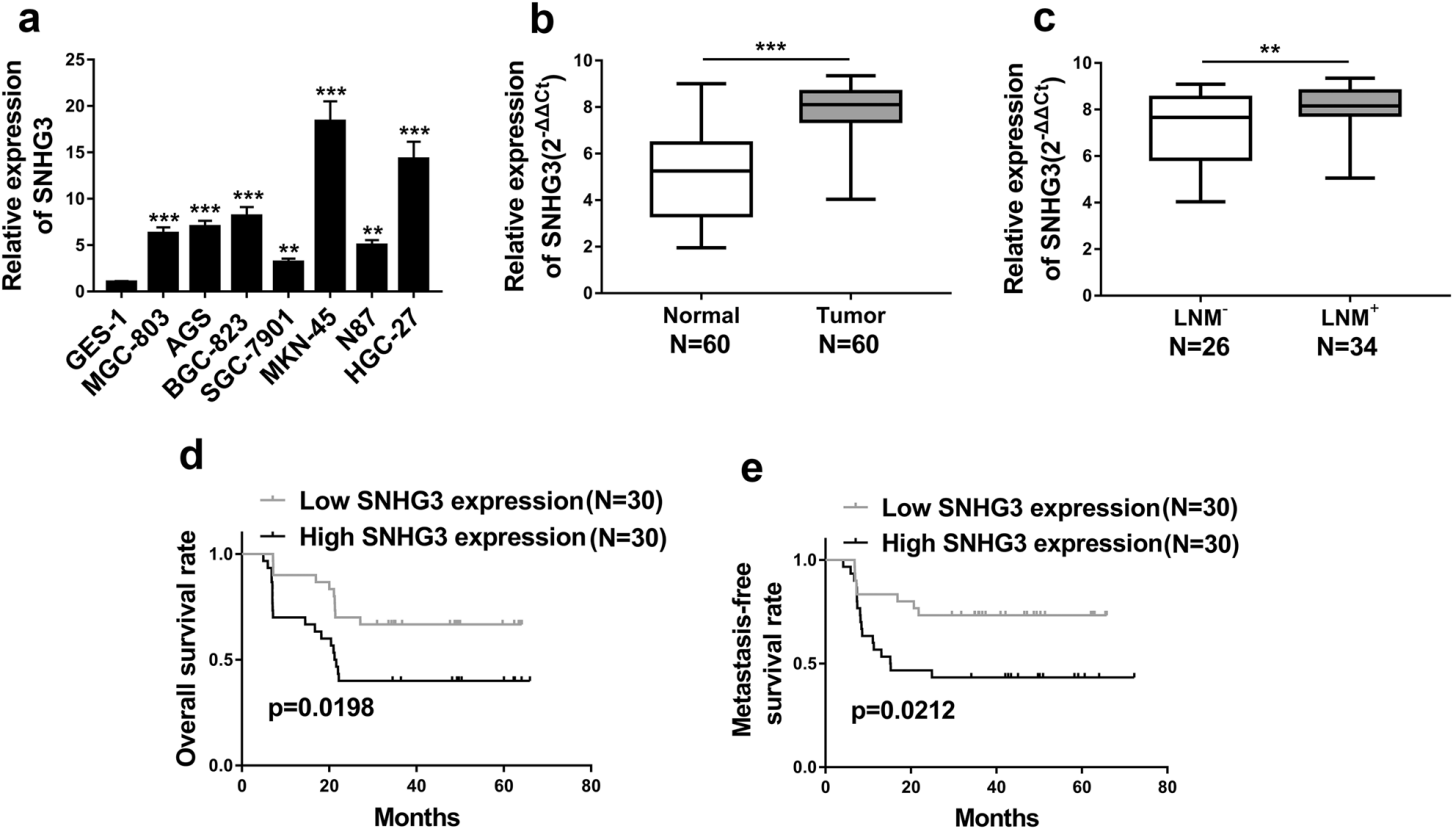
SNHG3 was highly expressed in GC and increased SNHG3 level was positively correlated with poor prognosis. **a** Expression of SNHG3 in various gastric cancer cell lines (MGC-803, AGS, BGC-823, SGC-7901, MKN-45, N87, HGC-27) compared to normal cell line GES-1 was detected by qRT-PCR. **P < 0.01; ***P < 0.001. **b** qRT-PCR assay was implemented to detect SNHG3 expression levels in 60 gastric cancer (GC) tissues (Tumor) and 60 non-tumor tissues (Normal), ***P < 0.001. **c** The SNHG3 expression levels were much higher in gastric cancer (GC) tissues of lymph node metastasis patients (LNM^+^) than patients without lymph node metastasis (LNM^–^) quantified by qRT-PCR analysis^.^ **P < 0.01. **d**, **e** Kaplan–Meier survival curves demonstrated that high levels of SNHG3 were associated with poor overall survival rate and metastasis-free survival rate. *P *<* *0.05*. Data are presented as mean ± SD and analyzed using independent samples *t*-test

We first observed significant overexpression of SNHG3 in cancer cell lines were observed in our cell panel including MGC-803, AGS, BGC-823, SGC-7901, MKN-45, N87 and HGC-27 (Fig. 1a). In addition, both CCK-8 and Transwell assays also consistently demonstrated that MKN-45 cells grew faster and was more invasive than SGC-7901 cells (Supplementary Fig. S1a and b). We further confirmed our preliminary in vitro results in clinical samples from GC patients. Consistent with the finding from cell culture, we noticed remarkable increase of SNHG3 in tumor in comparison with normal control in vivo as well (Fig. 1b). High level of SNHG3 also intimately associated with tumor progression as indicated by the aberrant abundance in the patients with lymph node metastasis compared to the metastasis-negative ones (Fig. 1c). Kaplan–Meier survival curves demonstrated that high SNHG3 level predicted an unfavorable clinical outcome in respect to both overall (Fig. 1d) and metastasis-free survival rate (Fig. 1e). Our results characterized the aberrant up-regulation of SNHG3 in GC and suggested a potential oncogenic role in this disease.

### SNHG3 promoted cell proliferation both in vitro and in vivo

**Fig. 2:**
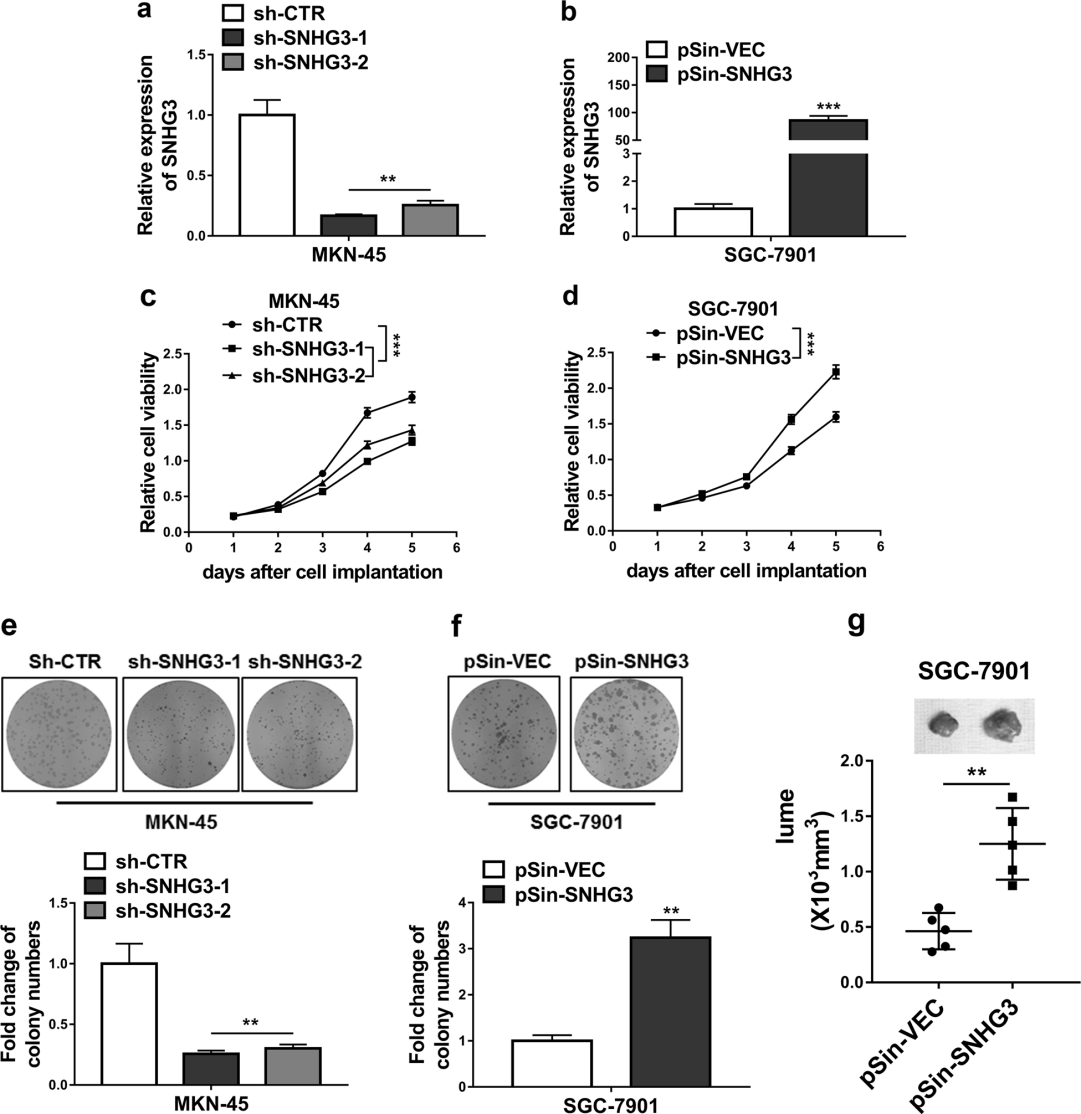
SNHG3 promoted GC cells proliferation both in vitro and in vivo. **a** RNA levels of SNHG3 were determined by qRT-PCR in MKN-45 cells stably transfected with SNHG3 shRNAs (sh-SNHG3-1 and sh- SNHG3-2) or empty vector (sh-CTR). **P < 0.01. **b** RNA levels of SNHG3 were determined by qRT-PCR in SGC-7901 cells stably transfected with SNHG3 plasmid (pSin-SNHG3) or empty vector (pSin-VEC). **c** CCK-8 assay was performed to evaluate cell proliferation of MKN-45 cells stably transfected with SNHG3 shRNAs (sh-SNHG3-1 and sh-SNHG3-2) or empty vector (sh-CTR). ***P < 0.001. **d** CCK-8 assay showing cell proliferation of SGC-7901 stably transfected with SNHG3 plasmid (pSin-SNHG3) or empty vector (pSin-VEC). ***P < 0.001. **e**, **f** Colony formation assay was conducted to assess cell proliferation of the same stable transfected MKN-45 and SGC-7901 cells. **P < 0.01. **g** Overexpression of SNHG3 promoted SGC-7901-derived tumor growth in xenograft model. **P < 0.01. Two-way ANOVA for c and d, student’s *t*-test for others

Next, we specifically established SNHG3-depleted cell line in MKN-45 and SNHG3-overexpressed cell line in SGC-7901. To exclude the potential off-target effects, two individual shRNAs were employed for SNHG3-silencing and ~80% and 75% knockdown efficiencies were achieved, respectively (Fig. 2a). The forced ectopic overexpression resulted in around 90-fold increase of SNHG3 in SGC-9701 cells (Fig. 2b). SNHG3-deficiency significantly compromised cell viability in MKN-45 cells (Fig. 2c), while SNHG3-proficiency remarkably enhanced cell viability in SGC-7901 cells (Fig. 2d). Furthermore, as shown in Fig. 2e, f, SNHG3-depletion greatly suppressed cell propagation in MKN-45 cells and ectopic introduction of SNHG3 significantly promoted cell proliferation in SGC-7901 cells. To eliminate possible artifacts involving in cell culture, we subcutaneously inoculated SGC-7901 into immunodeficient mice to evaluate the authentic effects of SNHG3 on tumor growth. In line with our observations in vitro, overexpression of SNHG3 markedly accelerated xenograft tumor progression in comparison with vector control (Fig. 2g). Therefore, we validated the pro-proliferation actions of SNHG3 in GC both in vitro and in vivo, which might underline its oncogenic properties in this disease.

### Knockdown of SNHG3 inhibited metastasis of GC cells both in vitro and in vivo

**Fig. 3:**
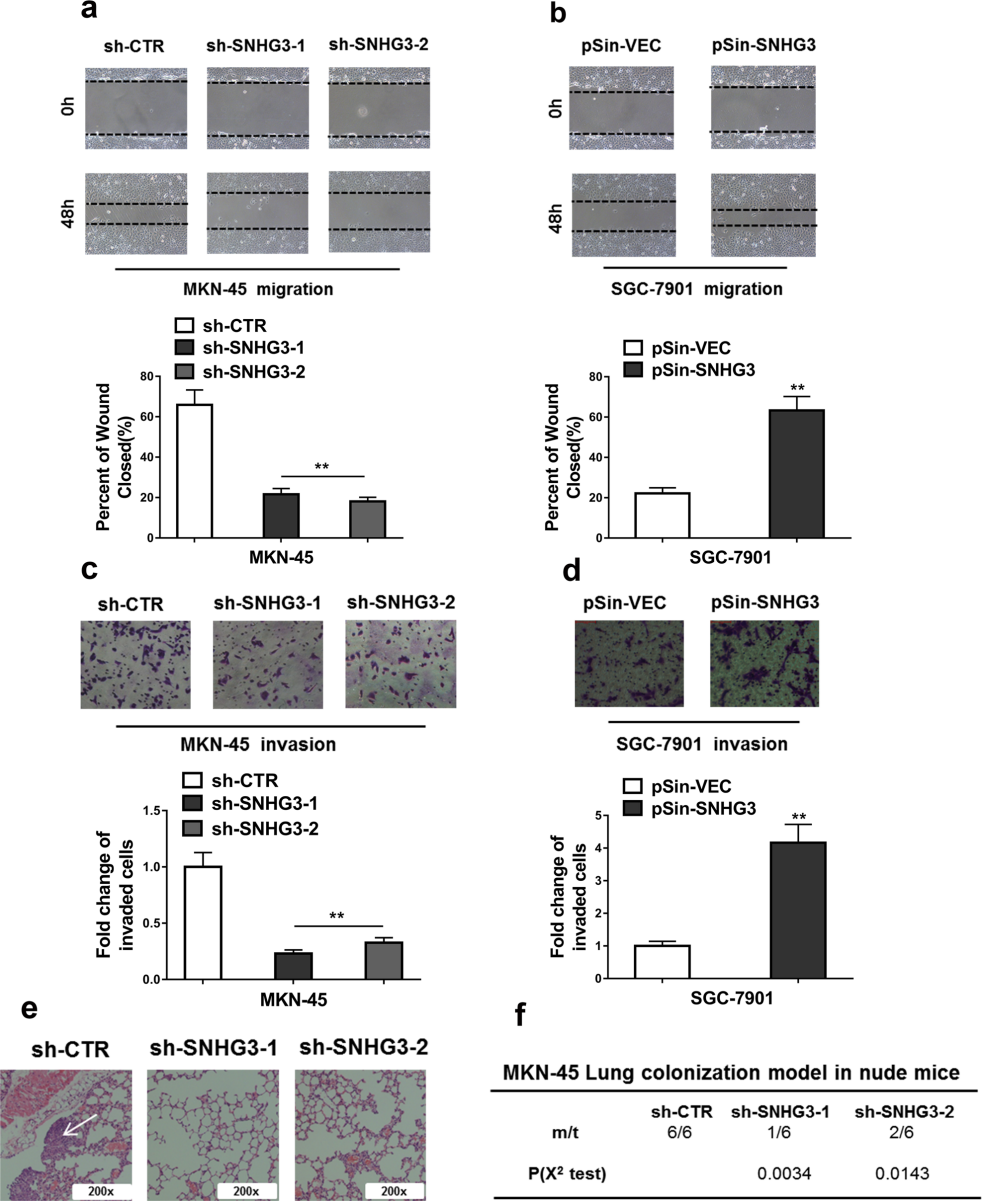
Knockdown of SNHG3 inhibited metastasis of GC cells both in vitro and in vivo. **a**, **b** Knockdown of SNHG3 significantly reduced, and overexpression of SNHG3 increased the migratory ability of GC cells (Wound healing assay). **P < 0.01. **c**, **d** Knockdown of SNHG3 significantly reduced, and overexpression of SNHG3 increased the invasive ability of GC cells (Transwell assay). **P < 0.01. **e**, **f** H&E staining of the metastatic nodules in the lung of MKN-45 cells which stably transfected with SNHG3 shRNAs (sh-SNHG3-1 and sh- SNHG3-2) or empty vector (sh-CTR) following tail vein injection into nude mice (×200 scale bars) and incidence of lung metastasis in mice following tail vein injection of the respective MKN-45 cells. *P < 0.05; **P < 0.01. χ^2^ test for *f*, student’s *t*-test for others

Next, migrative and invasive capacities of GC cells were determined in vitro by wound healing and transwell assay, respectively. As shown in Fig. 3a, SNHG3-deficiency significantly retarded the wound closure processing in MKN-45 cells. In sharp contrast, the ectopic SNHG3 remarkably accelerated the scratch healing in SGC-7901 cells (Fig. 3b). Likewise, the invasion capacity as indicated by the transwell assay was greatly compromised by SNHG3 depletion in MKN-45 cells (Fig. 3c) and enhanced by forced overexpression of SNHG3 in SGC-7901 cells (Fig. 3d). Furthermore, as shown in Fig. 3e, f, lung metastasis was significantly decreased in SNHG3-deficient MKN-45 cells in comparison with scramble control. Of note, SNGH3 also promoted EMT in GC cells (Supplementary Fig. S2). In addition, we employed xenograft models with SNHG3 interference in MKN-45 cells, and found knockdown of SNHG3 significantly inhibited the growth of MKN-45 cells-derived tumor (Supplementary Fig. S3a). Moreover, tail–veil injection model showed that overexpression of SNHG3 significantly promoted the lung metastasis of SNHG3-overexpressing-SGC-7901 cells (Supplementary Fig. S3b and c). Therefore, our data clearly demonstrated that SNHG3 contributed to the metastasis in GC.

### SNHG3 epigenetically regulated neighboring gene MED18 transcription by binding to EZH2

**Fig. 4:**
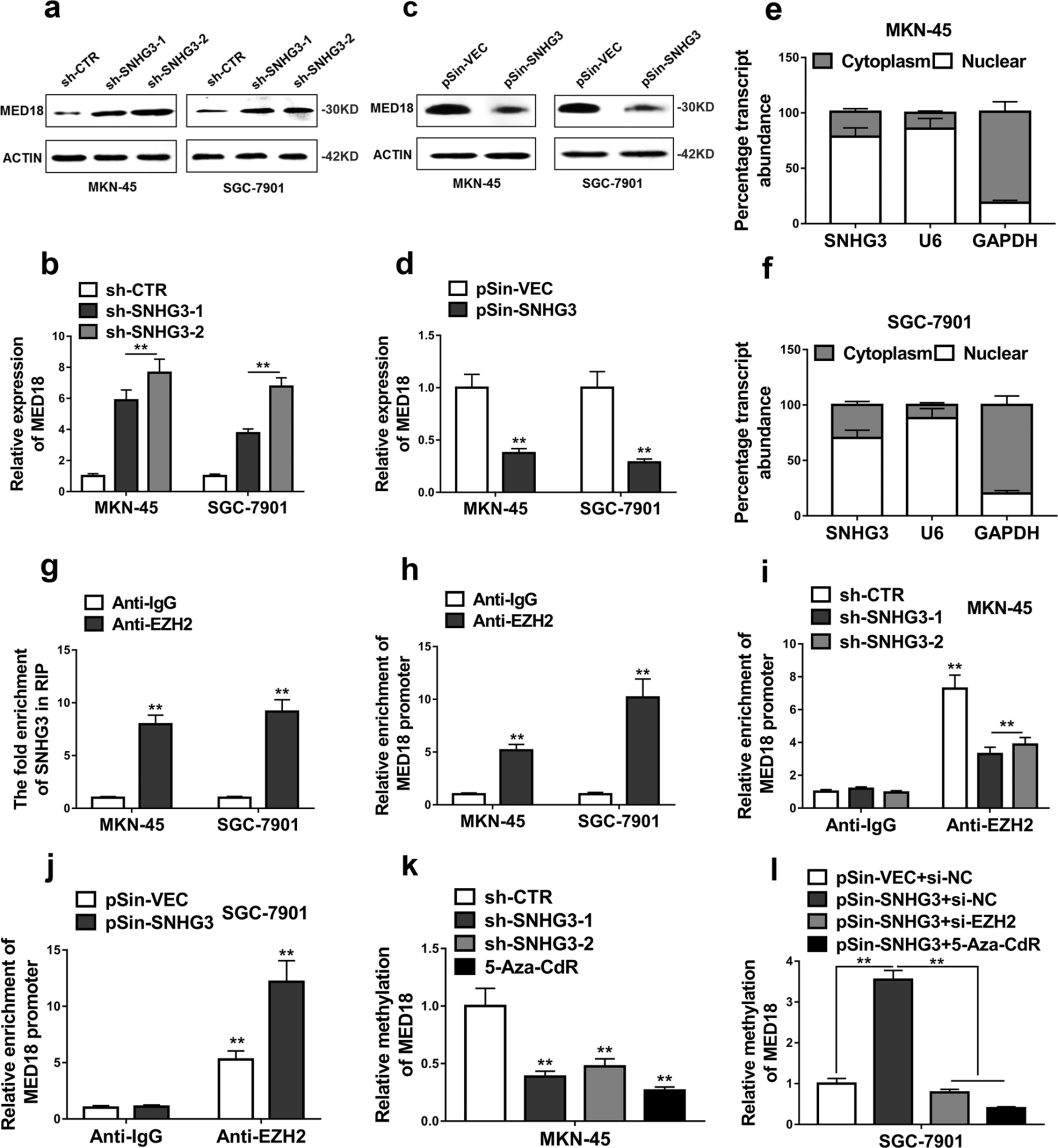
SNHG3 epigenetically regulated neighboring gene MED18 transcription by binding to EZH2. **a**–**d** Knockdown of SNHG3 significantly increased, and overexpression of SNHG3 suppressed the neighboring gene MED18 expression of GC cells detected by western blot and qRT-PCR analyses. **P < 0.01. **e**, **f** Relative SNHG3 expression levels in nuclear and cytosolic fractions of MKN-45 and SGC-7901 cells. Nuclear controls: U6; Cytosolic controls: GAPDH. **g** RIP assay was performed to determine the association between SNHG3 and EZH2. The fold enrichment of SNHG3 in MKN-45 and SGC-7901 cells with antibodies against EZH2 was relative to nonspecific IgG control. **P < 0.01. **h** ChIP-PCR assays were performed to determine the interaction between the promoter region of MED18 and EZH2 with antibodies against EZH2 and IgG. **P < 0.01. **i**, **j** ChI*P*-PCR assays of EZH2 enrichment of the promoter region of MED18 after the silence or overexpression of SNHG3 in GC cells with antibodies against EZH2. **P < 0.01. **k**, **l** The bisulfite sequencing PCR analysis (BSP) of the methylation levels of MED18 in SNHG3-downregulated. SNHG3-upregulated or 5-Aza-CdR-treated GC cells. **P < 0.01. The data represent the mean ± SD from three independent experiments. Student’s *t*-test

In view of the recognized mode-of-action that lncRNAs functioned via modulating neighboring genes^16,17^, we immediately analyzed the relative expression of SNHG3 neighbor gene, MED18. We first confirmed that relative expression of SNHG3 was suppressed in sh-SNHG3-SGC-7901 cells, whereas increased in pSin-SNHG3-MKN-45 cells (Supplementary Fig. S4a and b). Both protein and transcript level of MED18 was significantly increased by SNHG3 knockdown in MKN-45 and SGC-7901 cells (Fig. 4a, b). On the other hand, ectopic introduction of SNHG3 in both MKN-45 and SGC-7901 cells remarkably suppressed MED18 expression (Fig. 4c, d). To clarify the mode-of-action of SNHG3 in modulation MED18 expression, we further determined the cellular localization of SNHG3 in MKN-45 (Fig. 4e) and SGC-7901 (Fig. 4f) cells. We employed U6 and GAPDH transcripts as reference marks for nuclear and cytoplasm fractions, respectively. Our results unambiguously showed that the majority of SNHG3 transcripts were detected in nuclear instead of cytoplasm, which suggested the potential regulatory effects of SNHG3 on MED18 transcription processing. Next, we examined the potential interaction between SNHG3 and EZH2 using RIP assay and our results showed obvious enrichment of SNHG3 transcripts in the EZH2 immunoprecipitated complex compared with IgG control in both cell lines (Fig. 4g). The ChIP result further confirmed the direct binding of EZH2 on the MED18 promoter region, which indicated the epigenetic regulation on MED18 expression (Fig. 4h). SNHG3-deficiency evidently compromised the association of EZH2 with MED18 promoter in MKN-45 cells (Fig. 4i), which was greatly strengthened by SNHG3 overexpression in SGC-7901 cells (Fig. 4j). The methylation status of MED18 promoter was remarkably decreased by either SNHG3-knockdown or methyltransferase inhibitor, 5-Aza-CdR in MKN-45 cells (Fig. 4k). On the contrary, ectopic over-expression of SNHG3 stimulated the methylation level of MED18 promoter region in comparison with 5-Aza-CdR treatment (Fig. 4l). Of note, SNHG3 and EZH2 could simultaneously bind to MED18 promoter (Supplementary Fig. S5a and b). Also, we found SNHG3 modulation did not affect expression level of EZH2 (Supplementary Fig. S6). Our data uncovered the epigenetic modulation of MED18 expression by SNHG3 in GC cells.

### MED18 suppressed proliferation, migration and invasion of GC cells

**Fig. 5:**
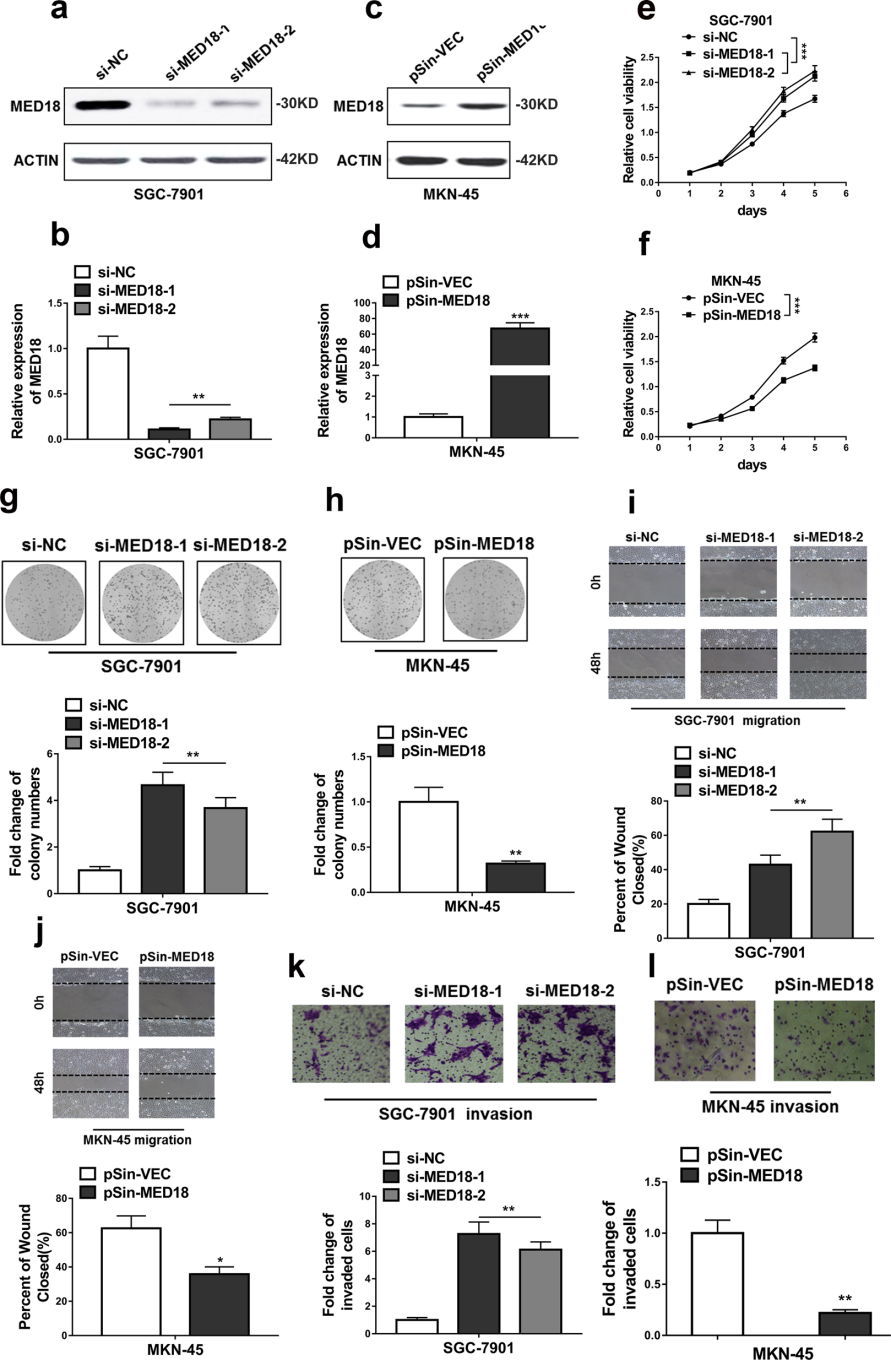
MED18 suppressed proliferation, migration and invasion of GC cells. **a**, **b** Protein and RNA levels of MED18 were determined by western blot and qRT-PCR in SGC-7901 cells transfected with MED18 siRNAs (si-MED18-1 and si-MED18-2) or empty vector (si-NC). **P < 0.01. **c**, **d** Protein and RNA levels of MED18 were determined by western blot and qRT-PCR in MKN-45 transfected with MED18 plasmid (pSin-MED18) or empty vector (pSin-VEC). ***P < 0.001. **e**, **h** CCK-8 assay and Colony formation assay showed knockdown of MED18 promoted and overexpression of MED18 inhibited cell proliferation of GC cells. **P < 0.01; ***P < 0.001. **i**, **j** Wound healing assay showed knockdown of MED18 promoted and overexpression of MED18 inhibited the migratory ability of GC cells. *P < 0.05; **P < 0.01. **k**, **l** Transwell assay showed knockdown of MED18 promoted and overexpression of MED18 inhibited the invasive ability of GC cells. **P < 0.01. The data represent the mean ± SD from three independent experiments. Two-way ANOVA for **e** and **f**, student’s *t*-test for others

Next, we sought to assess the predominance of MED18 in mediating the oncogenic actions of SNHG3 in GC cells. To this purpose, we first established MED18-deficient and -proficient stable cell lines in SGC-7901 and MKN-45 cells, respectively (Fig. 5a–d). MED18-deficiency remarkably improved the cell viability in SGC-7901 cells in comparison with control (Fig. 5e), while overexpression of MED18 greatly inhibited cell viability in MKN-45 cells (Fig. 5f). Likewise, MED18-depletion stimulated cell proliferation as shown by the colony formation assay in SGC-7901 cells (Fig. 5g) and forced MED18 overexpression suppressed cell proliferation in MKN-45 cells (Fig. 5h). Consistently, the wound closure was accelerated by MED18 knockdown in SGC-7901 cells (Fig. 5i) and delayed by MED18-expressing in MKN-45 cells (Fig. 5j). MED18-deficiency promoted cell invasion while MED18-proficiency inhibited this processing (Fig. 5k, l). Taken together, our results demonstrated the anti-tumor activities of MED18 in GC cells via suppression of cell proliferation, migration and invasion.

### SNHG3 promoted GC progression partly by regulating MED18 expression

**Fig. 6:**
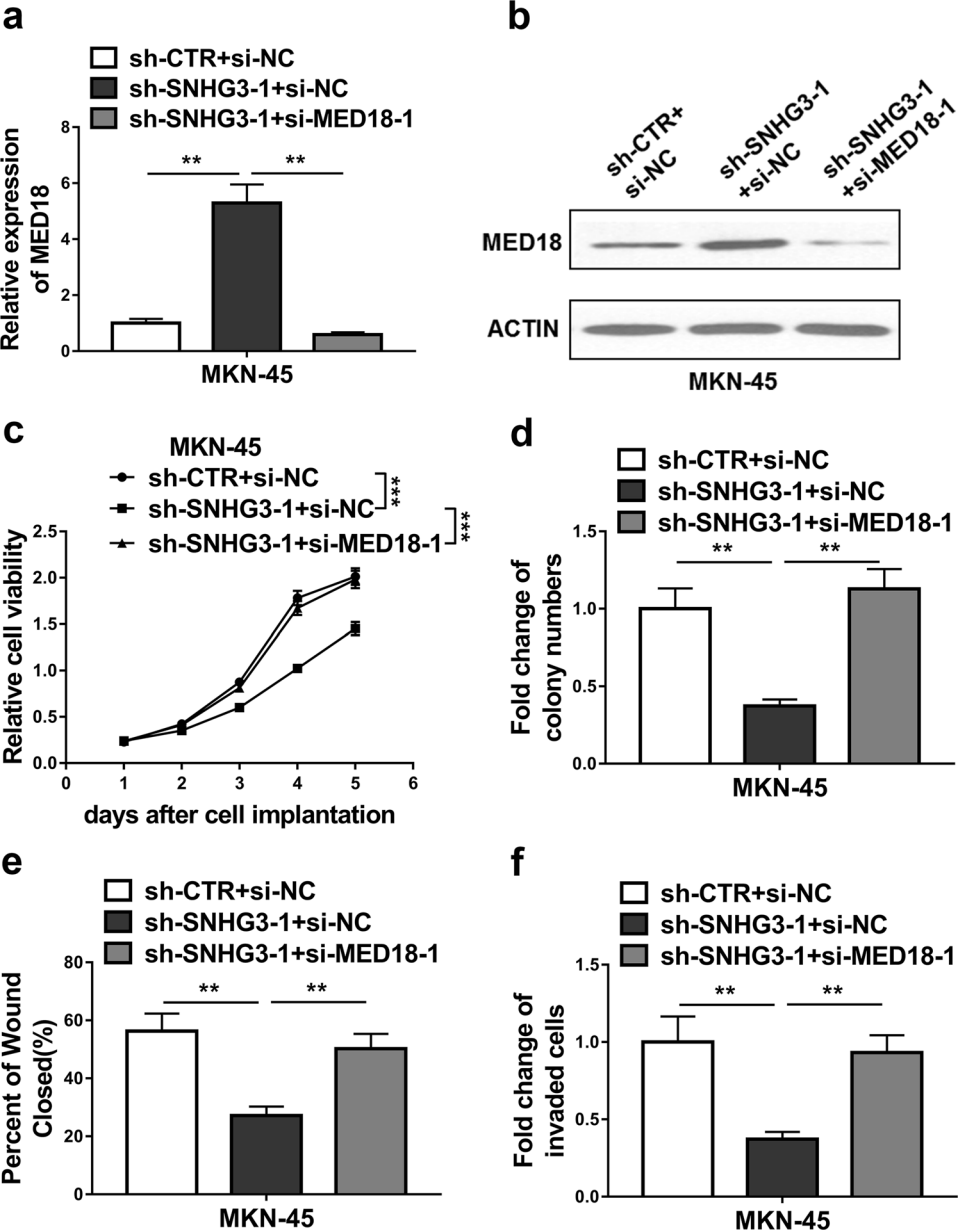
SNHG3 promoted GC progression partly by regulating MED18 expression. **a**, **b** The expression levels of MED18 were measured by qRT-PCR and western blot in the MKN-45 cells co-transfected with empty vector and negative control of siRNA(sh-CTR + si-NC), SNHG3 shRNA and negative control of siRNA(sh-SNHG3-1 + si-NC) or SNHG3 shRNA and MED18 siRNA(sh-SNHG3-1 + si-MED18-1). **P < 0.01. **c**, **d** CCK-8 assay and colony formation assay demonstrated that silence of SNHG3 inhibited MKN-45 cells growth, MED18 knockdown could rescue growth inhibition caused by SNHG3 knockdown in MKN-45 cells. **P < 0.01; ***P < 0.001. **e** Wound healing assay showed that silence of SNHG3 inhibited MKN-45 cells migration, MED18 knockdown could rescue migration inhibition caused by SNHG3 knockdown in MKN-45 cells. **P < 0.01. **f** Transwell assay showed that silence of SNHG3 inhibited MKN-45 cells invasion, MED18 knockdown could rescue invasion inhibition caused by SNHG3 knockdown in MKN-45 cells. **P < 0.01. The data represent the mean ± SD from three independent experiments. Two-way ANOVA for c, student’s *t*-test for others

To evaluate the dependence of oncogenic properties of SNHG3 on MED18 suppression in GC progression, we transiently silenced MED18 in SNGH3-deficient MKN-45 cells (Fig. 6a, b). Notably, MED18-silencing completely rescued the inhibition on cell viability imposed by SNHG3-deficiency in MKN-45 cells (Fig. 6c). Similarly, knockdown of MED18 restored the colony numbers in SNHG3-depleted cells (Fig. 6d). Furthermore, application of si-MED18 promoted the wound healing and stimulated cell invasion (Fig. 6e, f). In addition, in order to further verify SNHG3/MED18 functions in GC cells, we performed combination experiment in SGC-7901 cells, and the results showed MED18 overexpression impaired oncogenic behaviors afforded by overexpressing SNHG3 in SGC-7901 cells (Supplementary Fig. S7). Our data implicated that suppressed MED18 predominantly contributed to the oncogenic features of SNHG3 in GC progression in vitro.

## Discussion

In this study, we focused on the potential involvement of lncRNA SNHG3 in GC, which previously has been intensively investigated in lung cancer, liver cancer and ovarian cancer, whereas greatly underappreciated in GC. For the first time, we characterized the aberrant overexpression of SNHG3 in GC both in vitro and in vivo, which consolidated our primary finding and highlighted its critical relevance to clinical significance. The clinical data also suggested an unrecognized association between high SNHG3 and tumor metastasis, which was consistent with Zhang’s conclusion in hepatocellular carcinoma wherein SNHG3 induced EMT processing via modulating the miR-128/CD151 signaling^14^. The prognosis of patients with low SNHG3 manifested relatively favorable outcomes in comparison with high-SNHG3 group in respect to both overall survival and metastasis-free survival, which clearly indicated the potentially prognostic value of this lncRNA. We further experimentally demonstrated SNHG3-deficiency generated by shRNA knockdown significantly suppressed host cell viability and cell proliferation as indicated by colony formation capacity. In contrast, ectopic introduction of SNHG3 transcripts tremendously promoted both cell viability and cell proliferation, which underlined the oncogenic properties of SNHG3 in GC. Most importantly, this pro-tumoral phenotype was further confirmed in vivo with xenograft mice model. In addition, we experimentally validated the critical contributions of SNHG3 in tumor metastatic processing. SNHG3-silencing impaired cell migrative capacity as indicated by the delayed wound closure. The similar conclusion was drawn from transwell assay wherein ectopic overexpression of SNHG3 promoted cell invasion behavior in GC cells. Intriguingly, our tail-vein injection assay unambiguously demonstrated that SNHG3-silencing remarkably reduced the lung metastatic foci of MKN-45 GC cells. Mechanistically, we uncovered the relative expression of SNHG3-neighboring gene MED18 was inversely correlated with SNHG3 abundance in MKN-45 and SGC-7901 at both protein and transcript levels. Further characterization showed that the majority of SNHG3 transcripts localized in the nuclear compartment instead of cytoplasm, which implicated a potential transcriptionally regulatory action of this lncRNA. In line with the previous investigations showing the direct interaction between lncRNA and EZH2^18,19^, here we for the first time detected remarkable enrichment of SNHG3 in EZH2-immunoprecipitate from both MKN-45 and SGC-7901 cells. Along with SNHG3, EZH2 was clearly shown to bind to MED18 promoter region. Notably, our chromatin immunoprecipitation assay displayed that the enrichment of EZH2 on MED18 promoter was greatly compromised by SNHG3 knockdown, which consequently resulted in the decrease of methylation level and up-regulation of MED18. Our data suggested that the regulatory action of EZH2 on MED18 promoter critically depended on association with SNHG3. The predominance of MED18 in exerting oncogenic activity of SNHG3 was validated by the rescue assay as well, wherein co-knockdown of MED18 significantly reversed the tumor suppressing function elicited by SNHG3-silencing. In summary, our study for the first time uncovered the oncogenic properties of SNHG3 in GC and underlined the critical contributions of MED18 in this context, although the molecular mechanism underlying the aberrant overexpression of SNHG3 in GC was currently elusive.

Noteworthily, our data unambiguously uncovered the importance of MED18 downstream SNHG3 signaling in GC. MED18 was recognized as a component of the Mediator complex and co-activator for DNA-binding factors that activate transcription via RNA polymerase II, and the functioning relevance of MED18 consists in metabolism and regulation of lipid metabolism by peroxisome proliferator-activated receptor alpha (PPARalpha). Our study unraveled the novel epigenetic modulation of MED18 expression by SNHG3 along with EZH2. Although previous study proposed that MED18 played a negative role in transcription via the CDK/cyclin modul^20^, there were barely evidence in support of the mechanistic involvement of MED18 in the biology of human malignancies. To our best knowledge, our data firstly uncovered the tumor suppressor function of MED18 in GC, which might implicate a universal mode-of-action in other cancers and definitely worthy further investigations.

Our study also emphasized the critical contribution of EZH2 to the oncogenic actions of SNHG3 in GC. Following the increasingly recognized mode-of-action that lncRNAs are involved in assembly of epigenetic regulatory complexes, we employed RIP to demonstrate the direct binding between EZH2 and SNHG3. As an important member of the Polycomb-group (PcG) family, dysregulation of EZH2 via complexation with a range of lncRNAs has been disclosed in some human cancers. LncRNA-ANCR was first shown to regulate cell growth of osteosarcoma by interacting with EZH2 and subsequently affecting the expression of p21 and p27^21^. LncRNA-TUG1/EZH2 axis was characterized to promote EMT phenotype, cell proliferation and migration in pancreatic cancer through sponging miR-382^22^. Hu et al. provided evidence that HBx-upregulated lncRNA UCA1 promoted cell growth and tumorigenesis by recruiting EZH2 and suppressing p27Kip1/CDK2 signaling^23^. Han et al. proved that EZH2 promoted cell invasion and migration but not affecting cell proliferation by inhibiting E-cadherin, partly through association with MALAT-1 in pancreatic cancer^24^. LOC554202 was shown to modulate chordoma cell invasion and growth by recruiting EZH2 to epigenetically regulate miR-31 expression^25^. Plenty of data implicated the scaffold roles of macromolecular lncRNA in orchestrating the complex assembly of multiple factors in transcriptional regulation. In line with this notion, here we experimentally demonstrated that SNHG3 associated with EZH2 on the MED18 promoter, which consequently suppressed MED18 transcription and expression. In view of the universal involvement of lncRNAs in orchestrating the epigenetic function of EZH2, the next-generation sequencing of EZH2-binding RNA species would be necessary for more novel findings in this regard. Taken together, our investigations showed the SNGH3/EZH2-MED18 axis critically contributed to the malignant behaviors of GC, which might be further exploited for either prognostic or therapeutic purposes.

## Conclusions

In summary, our study uncovered the oncogenic properties of SNGH3 in GC and underlined the SNHG3/EZH2-MED18 signaling in this disease, which might hold unappreciated values for diagnostic purpose and therapeutic exploitation.

## Supplementary information

Supplementary Materials
