# Correction to: Long non-coding RNA SNHG3 promotes progression of gastric cancer by regulating neighboring MED18 gene methylation

**DOI:** 10.1038/s41419-022-04698-9

**Published:** 2022-03-11

**Authors:** Yi Xuan, Yanong Wang

**Affiliations:** 1 Department of Gastric Surgery, Fudan University Shanghai Cancer Center, Shanghai, 200032 China; 2 Department of Oncology, Shanghai Medical College, Fudan University, No 270 Dongan Road, Xuhui, Shanghai 200032 China

**Keywords:** Cancer, Cancer

Correction to: *Cell Death and Disease* (2019) 10:1–12 10.1038/s41419-019-1940-3, published online 18 September 2019

The original version of this article unfortunately contained a mistake in Fig. 3. The correct figure and figure legend can be found below. The authors apologize for the error.

**Fig. 3:**
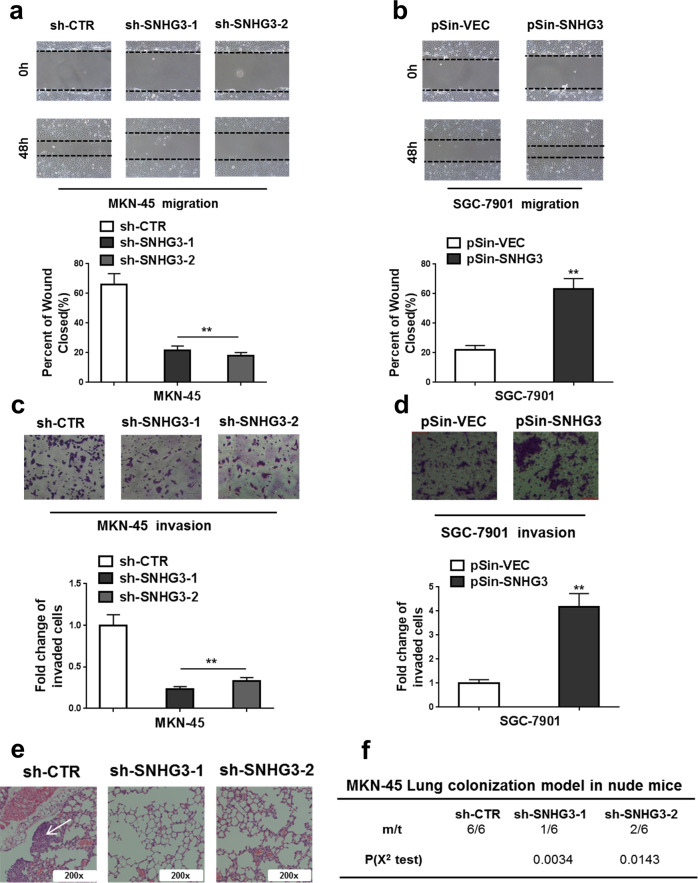
Knockdown of SNHG3 inhibited metastasis of GC cells both in vitro and in vivo. **a**, **b** Knockdown of SNHG3 significantly reduced, and overexpression of SNHG3 increased the migratory ability of GC cells (Wound healing assay). **P < 0.01. **c**, **d** Knockdown of SNHG3 significantly reduced, and overexpression of SNHG3 increased the invasive ability of GC cells (Transwell assay). **P < 0.01. **e**, **f** H&E staining of the metastatic nodules in the lung of MKN-45 cells which stably transfected with SNHG3 shRNAs (sh-SNHG3-1 and sh- SNHG3-2) or empty vector (sh-CTR) following tail vein injection into nude mice (200X scale bars) and incidence of lung metastasis in mice following tail vein injection of the respective MKN-45 cells. *P < 0.05; **P < 0.01. χ^2^ test for (**f**), student’s *t* test for others.

