# Supplementary material for: Long non-coding RNA SNHG3 promotes progression of gastric cancer by regulating neighboring MED18 gene methylation
Source: Cell Death Dis. 2019 Sep 18;10(10):694. doi: 10.1038/s41419-019-1940-3 (PMC6751301; doi:10.1038/s41419-019-1940-3)
Supplement: Supplementary file 1 — Supplementary Materials [file 41419_2019_1940_MOESM1_ESM.docx]

**Supplementary Materials**


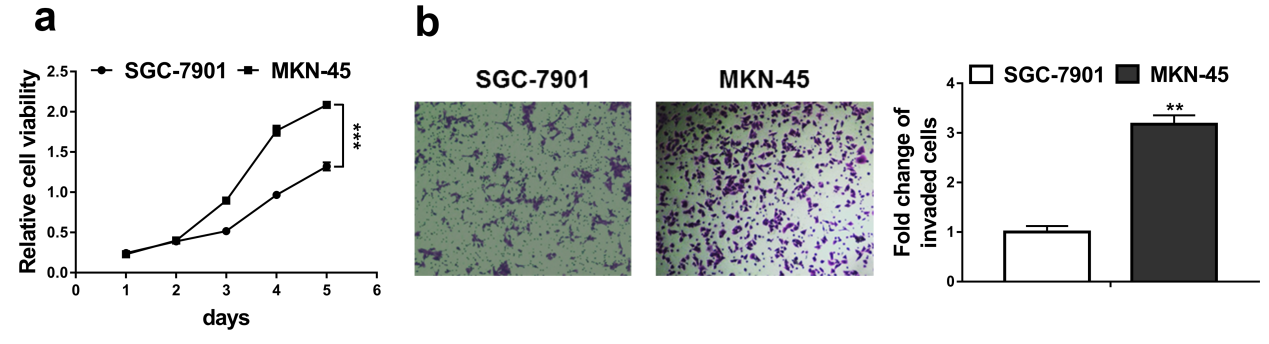


Fig. S1. (a) CCK-8 assay showing cell proliferation of SGC-7901 and MKN-45 cells. ****P*<0.001. (b) Transwell assay showing the invasive ability of SGC-7901 and MKN-45 cells. ***P*<0.01. The data represent the mean ± SD. Two-way ANOVA for a, student’s t-test for b.





Fig. S2. EMT markers (snai1, vimentin and E-cadherin) were detected in GC cells upon SNHG3 expression modulation by Western blot.


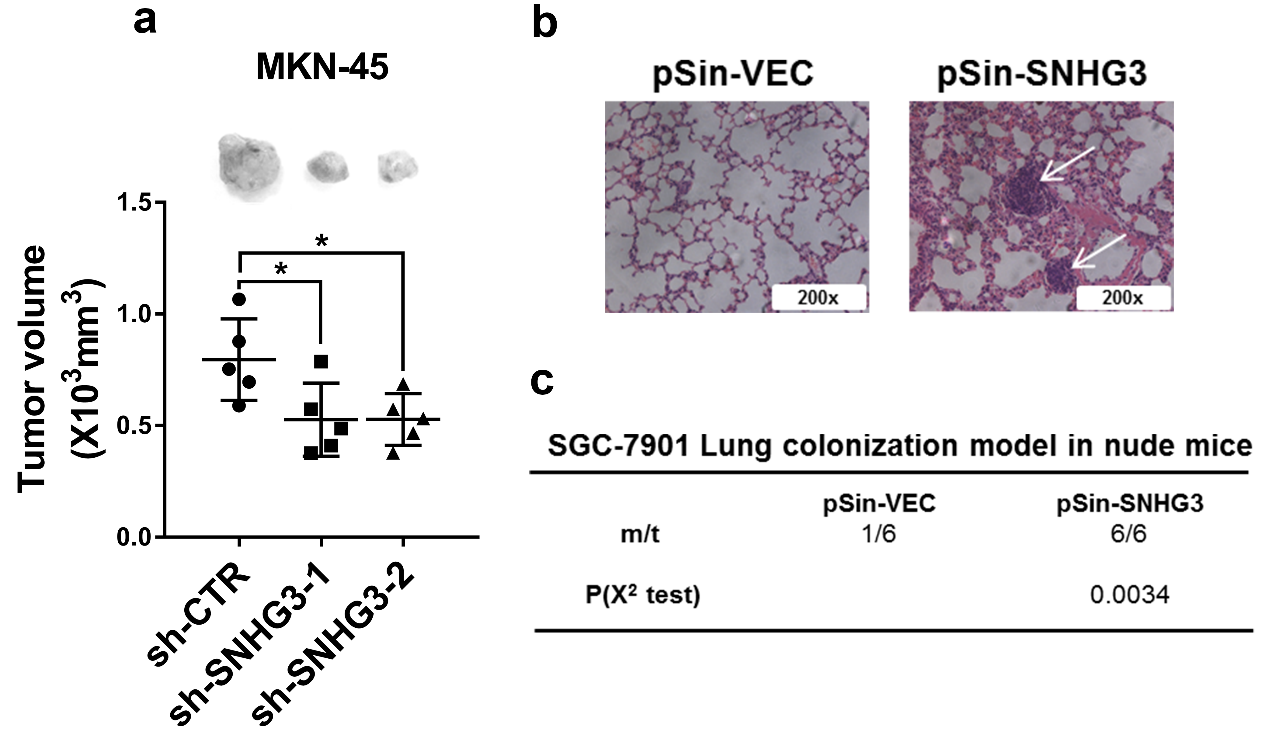


Fig. S3. (a) Knockdown of SNHG3 inhibited MKN-45-derived tumor growth in xenograft model. **P*<0.05. (b) H&E staining of the metastatic nodules in the lung of SGC-7901 cells which stably transfected with SNHG3 plasmid (pSin-SNHG3) or empty vector (pSin-VEC) following tail vein injection into nude mice (200X scale bars) and incidence of lung metastasis in mice following tail vein injection of the respective SGC-7901 cells. **P*<0.05; ***P*<0.01 (χ^2^ test for c, student’s t-test for others).


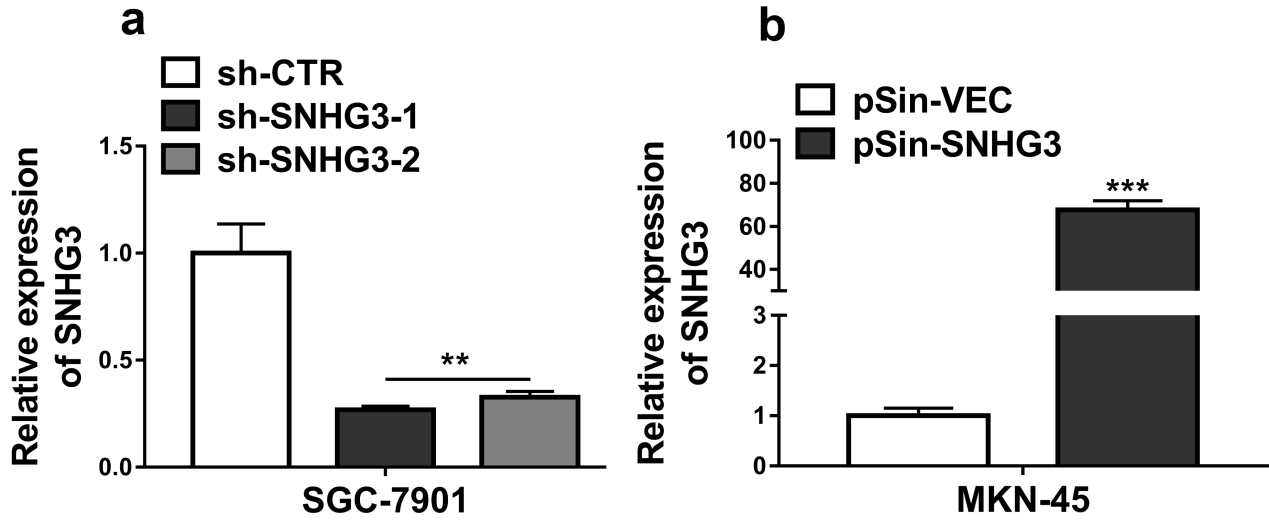


Fig. S4. (a) RNA levels of SNHG3 were determined by qRT-PCR in SGC-7901 cells stably transfected with SNHG3 shRNAs (sh-SNHG3-1 and sh- SNHG3-2) or empty vector (sh-CTR). ***P*<0.01. (b) RNA levels of SNHG3 were determined by qRT-PCR in MKN-45 cells stably transfected with SNHG3 plasmid (pSin-SNHG3) or empty vector (pSin-VEC). ****P*<0.001, student’s t-test.


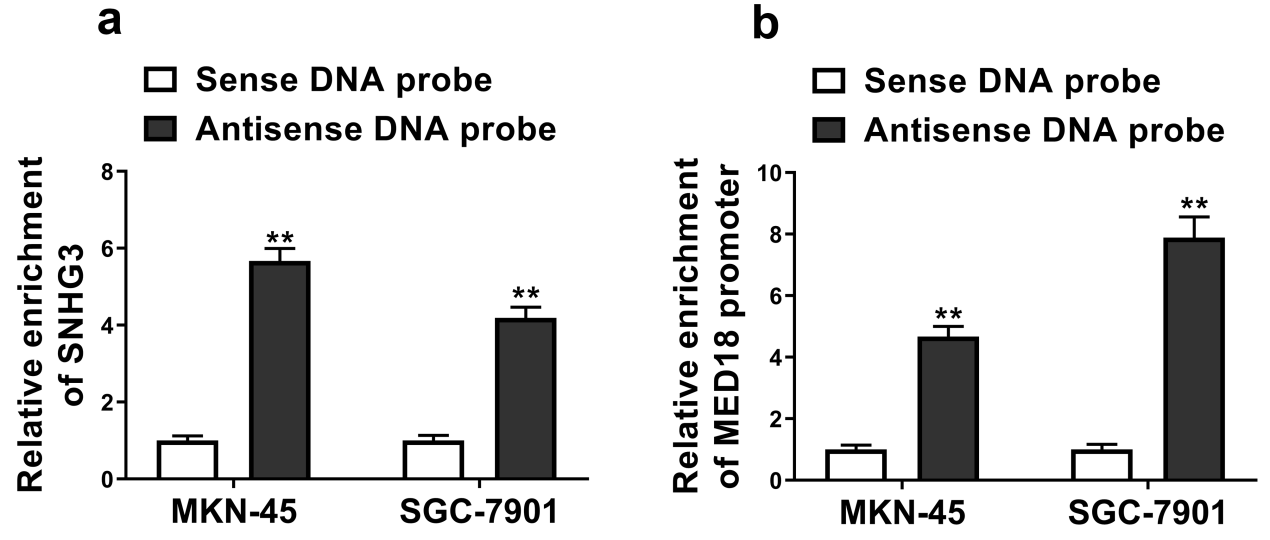


Fig. S5. (a and b) ChIP-coupled-RNA pulldown assay followed by qRT-PCR to detect the simultaneous presence of SNHG3 and EZH2 on MED18 promoter. Step 1, ChIP assay with anti-EZH2 antibody (anti-IgG antibody served as control) was used to enrich the EZH2 associated complex (RNA, DNA, protein). Step 2, biotin-labelled probes against SNHG3 was used to pulldown SNHG3 associated complex (RNA, DNA) from EZH2 associated complex in Step 1. Step 3, qRT-PCR was used to detected the levels of SNHG3 and MED18 promoter from SNHG3 associated complex in Step 2. ***P*<0.01, student’s t-test.





Fig. S6. (a-c) The expression level of EZH2 was detected by qRT-PCR and Western blot upon SNHG3 modulation in GC cells.





Fig.S7. (a and b) The expression levels of MED18 were measured by qRT-PCR and western blot in the SGC-7901 cells co-transfected with double empty vector (pSin-VEC+pSin-VEC), SNHG3 plasmid and empty vector(pSin-SNHG3+pSin-VEC) or SNHG3 plasmid and MED18 plasmid (pSin-SNHG3+ pSin-MED18). ***P*<0.01; ****P*<0.001. (c and d) CCK-8 assay and colony formation assay demonstrated that overexpression of SNHG3 promoted SGC-7901 cells growth, MED18 overexpression could rescue growth advantage caused by SNHG3 overexpression in SGC-7901 cells. ***P*<0.01; ****P*<0.001. (e) Wound healing assay showed that overexpression of SNHG3 enhanced SGC-7901 cells migration, MED18 overexpression could rescue migration advantage caused by SNHG3 overexpression in SGC-7901 cells. ***P*<0.01. (f) Transwell assay showed that overexpression of SNHG3 enhanced SGC-7901 cells invasion, MED18 overexpression could rescue invasion advantage caused by SNHG3 overexpression in SGC-7901 cells. ***P*<0.01. The data represent the mean ± SD. Two-way ANOVA for c, student’s t-test for others.
